# Integrating Nitrogen, Water, and Other Management Practices to Improve Grain and Ratoon Forage Yields in Perennial Rice

**DOI:** 10.3390/plants13223157

**Published:** 2024-11-10

**Authors:** Fuxian Xu, Dingbing Wang, Xingbing Zhou, Lin Zhang, Xiaoyi Guo, Mao Liu, Yongchuan Zhu, Hong Xiong, Changchun Guo, Peng Jiang

**Affiliations:** 1 Key Laboratory of Southwest Rice Biology and Genetic Breeding, Ministry of Agriculture and Rural Affairs, Rice and Sorghum Research Institute, Sichuan Academy of Agricultural Sciences, Deyang 618000, China; fuxianxu@scsaas.cn (F.X.); xingbingzhou@scsaas.cn (X.Z.); linzhang@scsaas.cn (L.Z.); guoxiaoyi@scsaas.cn (X.G.); maoliu@scsaas.cn (M.L.); yongchuanzhu@scsaas.cn (Y.Z.); hongxiong@scsaas.cn (H.X.); changchuns@scsaas.cn (C.G.); 2 Crop Ecophysiology and Cultivation Key Laboratory of Sichuan Province, Chengdu 611130, China; 3 Guzhang County of Agricultural and Rural Affairs, Xiangxi 416300, China; dingbingwang@scsaas.cn

**Keywords:** perennial rice, growth characteristics, regrowth rate, “mid-season rice–ratoon forage” cropping pattern

## Abstract

Perennial rice has recently garnered global attention due to its potential to save on seeds and labor costs and its high production efficiency. The “mid-season rice–ratoon forage” mode is a new planting system that has emerged in recent years. However, detailed information is still lacking on the regenerative characteristics, grain and ratoon forage yields, and forage nutrient content of perennial rice under different planting densities, nitrogen (N) rates, stubble heights, and water management practices. Four experiments with perennial rice were conducted in Sichuan Province, Southwest China, from 2017 to 2022. The results show that the rice grain and ratoon forage yields were significantly affected by year, planting density, and N. The grain yield was 28.18% and 60.81% lower in 2018F and 2019F, respectively, than in 2017F; similarly, the ratoon forage yield was 29.01% and 52.74% lower in 2018S and 2019S, respectively, than in 2017S. The low grain yield was mainly associated with lower numbers of spikelets per panicle and panicles per m^2^, which resulted from a lower regrowth rate, and the low ratoon forage yield was mainly attributed to the lower regrowth rate. The rice grain and ratoon forage yields increased with an increase in the N rate and planting density. The ratoon forage was found to be rich in crude protein, crude fat, crude fiber, calcium, nitrogen, phosphorus, potassium, and other nutrients. Moreover, the content of these nutrients increased significantly with an increase in the N rate. The regrowth rate and maximum tillers showed trends of first increasing and then decreasing with an increase in the stubble height under dry and wet alternation irrigation during the winter season. When the relative soil moisture decreased to below 80% during the winter season, the regrowth rate and seedling development index could reach more than 99% and 84%, respectively. Our results suggest that integrating N, water, and other management practices (including the combination of a 150 kg ha^−1^ N rate, 18.0 hills per m^2^, 10–20 cm rice stubble height, and alternating dry and wet irrigation during the winter season) is a feasible approach for achieving high grain and ratoon forage yields in perennial rice systems.

## 1. Introduction

Perennial rice can survive and be harvested several times in successive years after a single round of sowing and transplanting, enabling multi-year growth. Such rice crops can ratoon and regrow without additional sowing and transplanting after the first year. Previous studies have shown that the axillary buds on perennial rice nodes can overwinter, regrow, and develop into new plants in the following season [1,2], leading to lower agricultural inputs and a higher economic output [3]. The farming system for perennial rice is resilient to extreme environmental conditions and crop management practices, including extreme weather, storms, and water management [2]. Furthermore, compared with traditional annual transplanted rice, it saves seeds, reduces the need to raise rice seedlings and plow and harrow the paddy field, and reduces rice transplanting costs. On-farm survey results showed that perennial rice produced a yield of 7500–9000 kg ha^−1^ in each season and could save costs of approximately 1000 USD ha^−1^ each year, effectively addressing the labor shortage in rural areas and enhancing the economic benefits of rice production (unpublished data). These insights underscore the very broad applicability of overwintering rice.

Researchers have begun to develop perennial grains from a number of annual crops [4], and the potential benefits of perennial rice in sustainable farming systems are now attracting the attention of many agronomist scholars [5,6,7]. Many representative cultivars or strains of overwintering rice with numerous years of growth and harvest data have been screened or cultivated, including Nannu 501, Nanging 521, Tainong 67, Wuyunjing 7, DS89-1, Sugen 420, Dongye 1, and Yunda 107 [8,9,10,11,12,13]. Generally, these rice cultivars demonstrate strong cold tolerance. The perennial rice cultivar glutinous 89-1 exhibits strong cold tolerance in the germination and seedling stages [14]. Cold tolerance is controlled by multiple genes [15,16,17], and a QTL related to cold tolerance has been identified on chromosomes 4 and 8 [15,16]. Yield-related trait QTLs are largely controlled by additive–dominant inheritance with additional contributions from epistatic effects [18]. The grain yield of perennial rice is significantly influenced by root traits. Perennial rice has developed a robust root system that can absorb sufficient soil nutrients and water during the regrowth season and does not degenerate over successive regrowth seasons [19]. This resilience is supported by the regulation of rhizome development, which involves over ten genes [20]. The mechanism of overwintering contributes as follows: Under low temperatures and short-day conditions, there is an increase in the levels of ABA, which activates cold resistance genes and drives the synthesis of large amounts of cold resistance proteins. These proteins then contribute to the maintenance of membrane permeability, resulting in increased cellular cold resistance. Meanwhile, the expression of flower-forming genes is suppressed, and the synthesis of flowering substances is blocked due to low temperatures, which arrests plant development and leads to dormancy and overwintering survival [21].

Rice grain yield is influenced by genetic characteristics, environmental conditions, and crop management practices [22]. Previous studies have demonstrated that the yield of perennial rice is significantly impacted by the interaction between genotype and environment [23,24,25], and its high yield and stability are related to transplanting density and nitrogen management practices [26]. Although substantial progress has been made in breeding perennial rice cultivars for cold tolerance, with yields of over 9000 kg ha^−1^ in a single season [5], there is a lack of studies on the effects of high-yielding and efficient cultivation techniques on the yield and yield components of perennial rice, specifically those that consider planting density, nitrogen application rate, and water management practices. Unresolved issues, including uneven growth processes and lower regrowth rates during multi-season regeneration, result in lower yields after multiple seasons, which is why perennial rice has not been widely adopted. We hypothesize that the regenerative characteristics, grain and ratoon forage yields, and forage nutrient content of perennial rice can be improved by integrating nitrogen, water, and other management practices. To test this hypothesis, four field experiments were conducted in Luxian County, the primary region for ratoon rice production in Southwest China.

Our objectives are, first, to identify high-yield and efficient cultivation techniques based on the optimal N rate, planting density, and water management practices used during the winter season for the sustainable production of rice–ratoon forage in perennial rice systems. Another aim of this study, conducted from 2017 to 2022, is to characterize yield potential and the factors that determine yield in these systems. The results provide a scientific basis for the rapid and widespread promotion of perennial rice.

## 2. Results

### 2.1. Growth Duration of Perennial Rice

In experiment 1, the whole growth duration of the perennial rice was observed to be 9–11 days shorter in 2018F and 2019F than in 2017F (Table 1), but the full heading and maturity stages were delayed by 2–4 days and 9–14 days in 2018F and 2019F, respectively, compared with in 2017F. The growth duration was 5–6 days longer in 2018S and 2019S than in 2017S, and the full heading and maturity stages were delayed by 2–4 days and 6–9 days in 2018S and 2019S, respectively, compared with in 2017S.

### 2.2. Grain Yield and Yield Components of Perennial Rice

The grain yield of the perennial rice over successive seasons in the field in experiment 1 is shown in Table 2. The grain yield was significantly affected by the year (Y), N rate (N), and planting density (PD), with a significant interaction between Y and PD. In the first season (2017F: transplanting season), the average grain yield reached 8394.5 kg ha^−1^; however, it significantly decreased with each successive season of regrowth. The grain yield was 28.2% and 60.8% lower in 2018F and 2019F, respectively, than in 2017F. The grain yield in the first season of each year increased with the increase in the N rate and planting density. The grain yields were 3.5% and 2.0% higher under N_210_ than under N_90_ and N_150_, respectively. The planting density D_3_ resulted in a rice grain yield that was 18.1% and 10.0% higher than under D_1_ and D_2_, respectively, and 0.2% lower than under D_4_.

In subsequent regrowth seasons of the perennial rice, the regrowth rate and number of panicles per m^2^ decreased year by year following the first season of overwintering regeneration. There were relatively small differences in the grain filling and grain weight over the three years. The numbers of panicles per m^2^ and spikelets per panicle were significantly affected by the N rate (N), planting density (PD), and their interaction. However, N and PD and their interaction did not significantly affect the regrowth rate, grain filling, or grain weight. The number of panicles per m^2^ increased with the N rate, while the number of spikelets per panicle decreased with the increase in the N rate. The numbers of panicles per m^2^ were 11.0% and 4.4% higher under N_210_ than under N_90_ and N_150_, respectively. The pathway analysis results indicate that the number of panicles per m^2^ (X_2_) made the largest direct contribution to the grain yield, while the regrowth rate (X_1_) had the largest indirect effect on the grain yield (Table 3). Therefore, increasing the regrowth rate can significantly increase the number of panicles, which results in a high grain yield.

### 2.3. Forage Yield and Nutrient Composition of Overwintered Rice

In experiment 1, there was a significant extension of the perennial rice growth period, particularly the full heading stage of the second season in each year, which was delayed by more than 20 days compared with that of local conventional ratooning rice. As a result, the low temperature and low solar radiation during the grain filling stage were insufficient to meet the normal filling requirements, leading to a grain filling percentage of less than 30%. The values for grain plumpness and the grain plumpness index were only 31.1–39.8 and 8.3–11.8%, respectively (Table 4). Consequently, the ratoon crop harvested in the second season each year, which was nutritionally rich (Table 5), could only be utilized as animal feed. For the successive regrowth seasons of the perennial rice, the year (Y), N rate (N), planting density (PD), and the interactive effect of Y × N significantly affected the ratoon forage yield. On average, the ratoon forage yield was 40.9% and 111.6% higher in 2017S and 2018S, respectively, than in 2019S; additionally, it was 3.7% and 13.1% higher under N_150_ and N_210_, respectively, than under N_90_. Generally, the lowest planting density resulted in the lowest ratoon forage yield, and the highest planting density resulted in the highest ratoon forage yield. Compared with D_4_, the ratoon forage yield decreased by 13.2%, 2.7%, and 6.1% with the plant densities D_1_, D_2_, and D_3_, respectively.

The total N, crude protein, crude fat, crude fiber, calcium, phosphorus, and potassium levels in the ratoon forage were significantly affected by Y and N and also by PD, with the exception of phosphorus (Table 5). The interaction of Y × N had a significant effect on crude fiber and potassium. Similarly, the interaction of N × PD had a significant effect on crude fiber and calcium. The various nutrient contents of the ratoon forage decreased with each year of overwintering. A higher nitrogen application rate significantly increased the contents of multiple nutrients. The crude fiber, calcium, phosphorus, and potassium of the ratoon forage were significantly lower under N_90_ and N_150_ than under N_210_. At the same time, there were no significant differences between N_90_ and N_150_ in terms of the ratoon forage nutrient content. On average, the total N, crude protein, crude fat, crude fiber, and calcium were 2.7–4.1%, 4.2–5.2%, 6.4–9.2%, 1.5–2.1%, and 4.2–16.7% higher in D_3_ and D_4_, respectively, than in D_1_ and showed relatively small differences between D_3_ and D_4_. These results demonstrate that perennial rice can be grown using the new planting mode of “mid-season rice–ratoon forage” each year. Still, the problems of the low regrowth rate and poor maturity uniformity, which are exacerbated with each successive growing season of perennial rice, need to be addressed, as they adversely affect yield.

### 2.4. Effect of Stubble Height and Water Management on Overwintering Seedling Development

**Figure 1:**
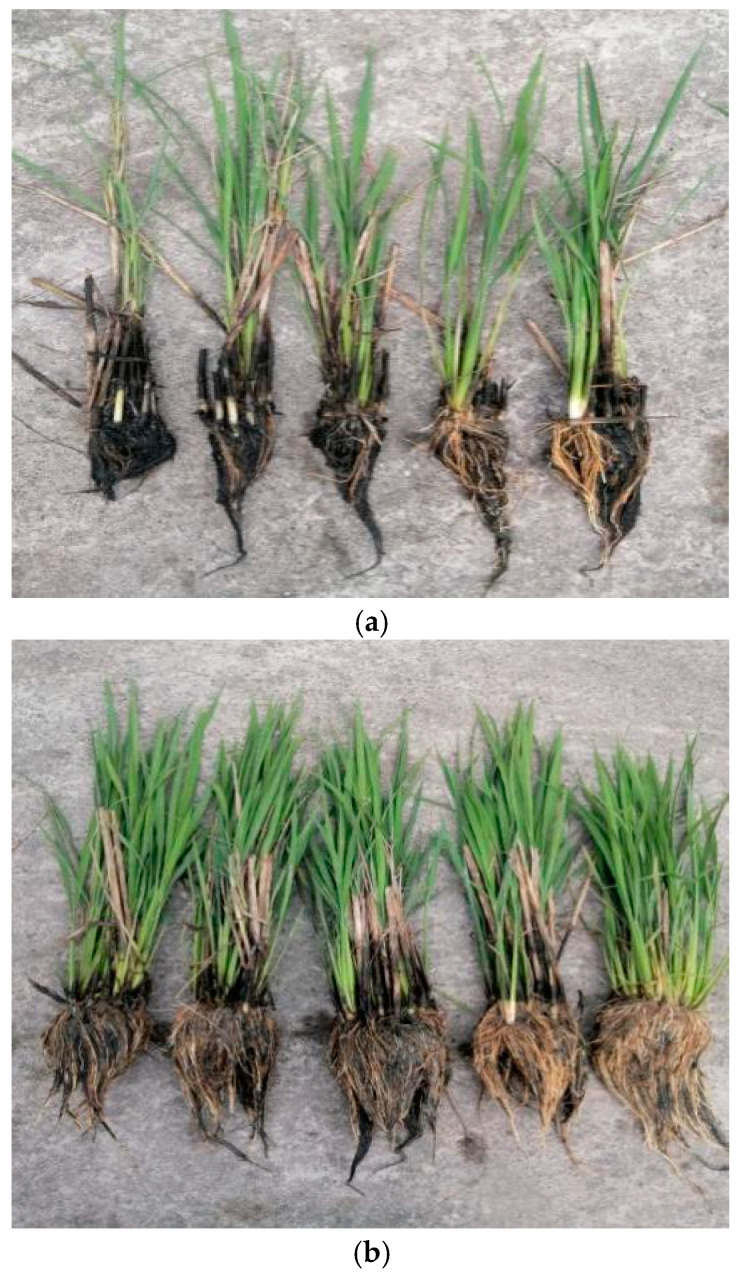
The development of regrowth seedlings of perennial rice grown during the winter season under two water management practices: (**a**) continuous irrigation and (**b**) drought management.

The regrowth rate and maximum tiller of the perennial rice over successive seasons in the field in experiment 2 are shown in Table 6. The regrowth rate and maximum tiller were significantly affected by the year (Y), water management practice (W), stubble height (H), and their interactions; however, the interaction of Y × H had no effect on the regrowth rate. The growing point of regenerated seedlings in perennial rice is mainly concentrated in the aboveground basal internode; when the stubble height is 0 cm (flat mud cut), the growing point of the regenerated seedling is cut off. As a result, the regrowth rate is 0 regardless of the water management practice. Under shallow irrigation, the regrowth rate was as high as 73.35–82.51% for a 10–40 cm stubble height, with no significant differences across the various stubble heights. The regrowth rate was generally slightly higher under the 30 and 40 cm treatments than under the 10 and 20 cm treatments. However, the maximum tillers per hill were 32.1–49.6% higher under the 10 and 20 cm treatments than under the 30 and 40 cm treatments. Under alternating wet and dry irrigation, the regrowth rate reached 100% in the 10 and 20 cm treatments, and it exceeded 94% in the 30 and 40 cm treatments. The maximum tillers per hill were significantly higher under the 10 and 20 cm treatments than under the 30 and 40 cm treatments. On average, the regrowth rate and maximum tillers per hill were 31.9–65.1% and 18.9–20.5% higher under alternating wet and dry irrigation than under shallow irrigation, respectively. Thus, it appears that the presence of a water layer in winter results in a large amount of root death, which is likely the main reason for the high death rate of seedlings during regrowth (Figure 1).

### 2.5. Effect of Soil Moisture Content on Overwintering Seedling Development and Uniformity

The first-season rice grain yield and second-season ratoon forage yield of the perennial rice were significantly affected by the year (Y), drought treatment (D), and their interaction (Table 7). Each year, the first-season rice grain yield and second-season ratoon forage yield of the perennial rice varied with the soil moisture content. Regarding the different drought treatments, D_30_, D_60_, D_90_, and D_120_ significantly increased the first-season rice grain yield compared with the control (CK), with increases of 34.4–34.6%, 58.0–28.4%, 74.7–80.0%, and 73.4–78.5%, respectively; similarly, the same treatments significantly increased the second-season ratoon forage yield compared with CK, with increases of 4.0–6.2%, 16.8–21.8%, 27.3–33.3%, and 28.3–38.0%, respectively. The first-season rice grain yield and second-season ratoon forage yield were significantly higher under D_90_ and D_120_ than under D_30_ and D_60_. In contrast, there were relatively small differences between D_90_ and D_120_ regarding the first-season rice grain yield and second-season ratoon forage yield.

Under the drought treatment, the regrowth rate, sprouting bud stem rate, and seedling development index increased with the treatment duration and were significantly higher than under CK. The regrowth rate, sprouting bud stem rate, and seedling development index under D_90_ and D_120_ reached 99.34–100%, 85.44–91.92%, and 84.83–91.92%, respectively, and they were significantly higher than those under D_30_ and D_60_. The time from the initiation of heading to full heading was approximately 6.6–7.5 days shorter under D_90_ and D_120_ than under CK, indicating that the winter drought was beneficial for improving the growth characteristics of the perennial rice. There were relatively small differences between D_90_ and D_120_ in terms of the regrowth rate, sprouting bud stem rate, seedling development index, and days from the initiation of heading to full heading. These results indicate that a low soil water content during the overwintering period is conducive to improving root vigor, which enhances the regrowth rate and shortens the heading stage, thereby increasing the first-season rice grain yield and second-season forage yield. The results also demonstrate that when the relative soil water content is reduced below 80%, the regrowth rate and seedling development index can reach above 99% and 84%, respectively; thus, this value can be used as a threshold in the water management of perennial rice fields.

### 2.6. Integrated Technique for Perennial Rice in Field Demonstration

The first-season rice grain yield, yield components, and second-season ratoon forage yield of the perennial rice in the field in experiment 3 in the year 2022 are shown in Table 8. The regrowth rate, number of panicles per m^2^, and number of spikelets per panicle were 41.1%, 23.25%, and 3.5% higher under the overwintering whole seedling technique (OWST) than under CK_1_, respectively. As a result, the OWST resulted in a 30.9% and 33.0% higher first-season rice grain yield and second-season ratoon forage yield, respectively, than the CK_1_ treatment. The first-season rice yield and the second-season forage yield were the same under the OWST and CK_2_. The OWST has attracted considerable attention because it saves seeds, seed beds, and labor costs. The OWST has a significant enhancement effect and can be adapted to local conditions.

## 3. Discussion

### 3.1. Production Problems and Crop Management Countermeasures for Perennial Rice

Research on perennial rice has been conducted for over 60 years, mainly focusing on the selection of cold-tolerant varieties, genetic principles [5], gene localization [15,16,17,18,20], cold tolerance mechanisms [18], and genotype–environment interactions [23,24]. However, few studies have been conducted on the impact of crop management practices on the grain yield and yield components of perennial rice. Zhang et al. [26] conducted a field experiment with four N rates and three plant densities to assess and ameliorate the adverse effects of N fertilizer on the regenerative ability of perennial rice across successive regrowth seasons. Their results showed that applying 180 kg ha^−1^ with 22.6 hills per m^2^ resulted in a stable and high grain yield by enhancing the regrowth rate, N productivity, and root activity while optimizing yield components (such as the number of panicles and number of spikelets per panicle) in the perennial rice system. In this study, the regrowth rate, first-season grain yield, and second-season ratoon forage yield significantly decreased with each successive season of perennial rice regrowth (Table 2 and Table 5). However, the grain and ratoon forage yields increased with the increase in the N rate and planting density. Our results indicate that an increased N rate and planting density (2017F: transplanting season) could alleviate the negative effects of successive regrowth on the regenerative ability, grain yield, and ratoon forage of perennial rice. This suggests that a management practice integrating the nitrogen application rate and planting density can be used to enhance the rice grain and ratoon forage yields by improving the regrowth rate and optimizing the yield components.

The higher grain yield is mainly attributed to the higher regrowth rate and number of panicles per m^2^ (Table 2). The better root characteristics under alternating wet and dry irrigation were partly responsible for the higher regrowth rate and maximum tiller number (Table 6 and Figure 1). In general, root activity and root biomass accumulation under alternating wet and dry irrigation are higher than under continuous irrigation [27], and large root dry matter with high root activity implies a strong water and nutrient absorption capacity [28,29], which tends to favor high regrowth rate and rice grain production. In the present study, the regrowth rate, first-season grain yield, and second-season ratoon forage yield were significantly higher under the drought treatment during the perennial rice’s overwintering period than under continuous irrigation (Table 7). These findings suggest that the regrowth rate, first-season grain yield, and second-season ratoon forage yield could be further improved by optimizing the rice stubble height, water irrigation, and days of drought treatment during perennial rice’s overwintering period. Accordingly, our results indicate that the following combination can be used to enhance the grain and ratoon forage yields of perennial rice: an N rate of 150 N kg ha^−1^, a planting density of 18.0 hills per m^2^, a stubble height of 10–20 cm, and winter dry or dry–wet alternating water management. Through this new integrated crop management practice, the key problems of the low regrowth rate and yield potential of perennial rice have been resolved. In our field demonstration, the grain and ratoon forage yields were more than 30% higher when applying the OWST than when applying local overwintering technology (CK_1_) and were comparable to the yield obtained for the local conventional technique (CK_2_). Our results suggest that the rice grain and ratoon forage yields of perennial rice can be easily enhanced using a strategy based on integrating N, water, and other management practices.

### 3.2. Suitable Areas for the “Mid-Season Rice–Ratoon Forage” Planting Mode in the Perennial Rice System

**Figure 2:**
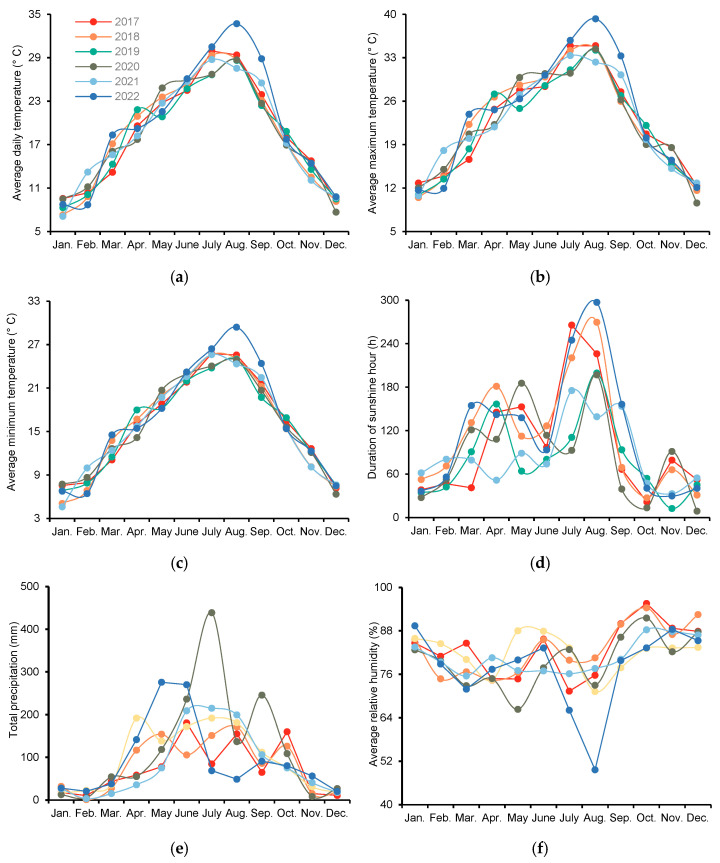
Average daily temperature (**a**), average maximum temperature (**b**), average minimum temperature (**c**), duration of sunshine hours (**d**), total precipitation (**e**), and average relative humidity (**f**) from 2017 to 2022. Each data point represents the mean/sum value of each month.

Previous research has demonstrated that the diversity in perennial rice-planting patterns is influenced by the physiological characteristics of different perennial rice varieties and the local ecological conditions. The new perennial rice variety “Yunda 107” can be harvested twice a year in dry and hot rice-planting areas such as the Red River Basin in Yunnan, with the early-season rice grain yield exceeding 9000 kg ha^−1^ [13]. The study location has a tropical monsoon climate, and the annual rainfall and average temperature are 1136.6 mm and 23.3 °C, respectively. The rice line 4020, selected by [9], can safely overwinter in Chengdu at an average temperature of 5.4 °C for January and an extreme low of −7.6 °C, and it can be harvested three to five times a year, yielding 2000–5000 kg ha^−1^ of rice each harvest. In our present study, the average maximum, minimum, and daily temperatures in January were above 10.0, 4.5, and 7.0 °C, respectively (Figure 2). The first-season grain yield and ratoon forage yield of the rice cultivar Lunuo 1 were 3289.40–8394.49 kg ha^−1^ and 2676.64–5663.78 kg ha^−1^, respectively (Table 2 and Table 5). Compared with CK, D_90_ and D_120_ significantly increased the first-season rice grain yield by 74.7–80.0% and 73.4–78.5%, respectively, and the second-season ratoon forage yield by 27.3–33.3% and 28.3–38.0%, respectively (Table 7). The OWST also resulted in a 30.9% and 33.0% higher first-season rice grain yield and second-season ratoon forage yield, respectively, than the CK_1_ treatment (Table 8). This finding indicates that the rice cultivar Luonuo 1 can safely overwinter in Lu County, and optimizing the nitrogen, water, and stubble height management practices could further improve the first-season rice grain yield and second-season ratoon forage yield of perennial rice.

In Lu County, located in southeastern Sichuan Province, below an altitude of 350 m, “mid-season rice–ratoon rice” is the main planting pattern. Lu County is the largest ratoon rice-producing county in Sichuan Province, with an annual planting area of approximately 33,000 hectares, accounting for 10% of the total ratoon rice-planting area in Sichuan. In this region, which has a subtropical humid climate, the yield of mid-season hybrid rice is 8500–10,000 kg ha^−1^ and the yield of ratoon rice is 1800–3000 kg ha^−1^ using the “mid-season hybrid rice–ratoon rice” system [30]. However, the “mid-season hybrid rice–ratoon rice” system requires greater productivity inputs each year for tillage, raising seedlings, and seedling transplanting. Each year, the maturity of the first-season perennial rice was delayed by 9–14 days compared with the local hybrid mid-season rice (Table 1); the temperature and light conditions were insufficient to meet the requirements for heading and grain filling in the second season. Moreover, each year, the harvest date of the second-season ratoon rice was delayed by over 30 days compared with that of the local traditional regenerated rice. This is because the seedlings germinate from the upper root in conventional ratooning rice [31,32] and from the underground stem nodes in the perennial rice system [9]. The average temperature during grain filling was lower than required, and only early-heading grains could be filled normally. The average grain plumpness index was only 8.29–11.83% (Table 4). Therefore, under the ecological conditions of this region, perennial rice can be used in a “mid-season rice–ratoon forage” system, in which ratoon forage grass is rich in crude protein, crude fat, crude fiber, calcium, nitrogen, phosphorus, potassium, and other nutrients. The results for these nutrient content parameters indicate that ratoon rice is a good-quality forage. Similarly, the rice cultivar Zhunliangyou 608 could be used as ratoon rice in subtropical and temperate rice-planting areas to produce good-quality forage, and a lower cutting height for ratoon rice could enhance ratoon forage yield and feeding quality [33]. In the present study, the content of these nutrients increased significantly with increases in the nitrogen application rate and planting density. Moreover, under integrated nitrogen, water, and stubble height management practices, the “mid-season rice-ratoon forage” system could achieve an annual yield of 8500 kg ha^−1^ of mid-season rice and 7300 kg ha^−1^ of dry weight of ratoon forage grass (Table 8). The results suggest that both mid-season rice grain yield and ratoon forage yield could be simultaneously improved by optimizing the nitrogen, water, and stubble height management practices. Moreover, the “mid-season rice–ratoon forage” mode is suitable and can thus be promoted for use in terraced areas with guaranteed water sources, as it can alleviate issues associated with winter forage shortages in the development of the local storage and grazing industry. 

### 3.3. Issues to Be Addressed by Future In-Depth Studies 

The first issue is that only a few perennial rice varieties can be promoted and applied, mainly those within the japonica rice [10,12,13] and indica glutinous rice [5] subspecies. The main planting areas suitable for the promotion of perennial rice are in regions with better light and heat resources in southern China, such as in the southeastern Sichuan Basin. Here, the annual temperatures and light and heat resources meet the requirements for the “mid-season rice–ratoon rice” cropping system; however, the climate during the grain filling period of the main mid-season rice crop is characterized by frequent drought, low light, high humidity, and high temperatures. These unfavorable climatic conditions result in poor rice quality, a lower head rice rate, and a high rate and degree of chalkiness of the rice grains of the main mid-season rice crop [34,35]. Moreover, promotion of the perennial rice varieties Yunda 107 and Lunuo 1, already characterized by poor rice quality, in regions with high temperatures further reduced their rice quality. In general, these adverse effects of high temperatures on rice quality could be alleviated by shifting or delaying the full heading stage and prolonging the grain filling period of mid-season hybrid rice. In this study, the full heading date was delayed by 2–4 days in 2018F and 2019F compared with in 2017F, and the grain filling period was 9–14 days longer in 2018F and 2019F than in 2017F (Table 1). To enable adaptation to these unfavorable climatic conditions, it is necessary to select and breed indica–japonica hybrids with better rice quality, lower temperature resistance at the seedling stage, increased high-temperature resistance at the heading stage, and a shorter growth duration. 

The second issue pertains to the cycle suitable for perennial rice cultivation. Overwintering perennial rice has the advantage of greatly reducing production costs, but it is accompanied by an increase in the number of planting years and successive growth seasons, in which the yield will gradually decline. Therefore, identifying an annual cycle suitable for multiple harvests is essential. The results of this study demonstrate that it is feasible for Lunuo 1 to be planted for six successive growing seasons. However, the optimal regrowth cycle for other different perennial rice varieties under different ecological conditions should be studied according to local conditions. 

Regarding the third issue, moisture management throughout the overwintering period is the key crop management practice determining the success or failure of perennial rice. Our results suggest that, during the perennial rice overwintering period, adopting moderate drought and maintaining a relative soil moisture content of 68–79% are beneficial for improving root activity, the regrowth rate, the sprouting bud stem rate, and the seedling development index, which result in a high rice grain yield; additionally, this relative soil moisture content value can be used as the threshold for moisture management in perennial rice paddy fields during the overwintering period. However, the physiological mechanisms of perennial rice and their effects on yield still require further exploration.

## 4. Materials and Methods

### 4.1. Experimental Design and Performance

Field experiments were conducted with the perennial rice cultivar Lunuo 1 in Lu County (29°6′ N, 105°6′ E, 300 m asl), Sichuan Province, China. Lu County is located in the hilly region of the Southwest Sichuan Basin, which has a humid subtropical climate, and rice is generally harvested twice a year (in the main season and the ratoon season). The climatic conditions are shown in Table 9. In general, the average daily temperature, average maximum temperature, average minimum temperature, and duration of sunshine hours exceed 18.0 °C, 22.0 °C, 15.0 °C, and 980 h, respectively.

#### 4.1.1. Experiment 1: Effect of Planting Density and N Rate on Grain Yield of Perennial Rice

A fixed field experiment employing a split-plot design was conducted with three replicates over six successive seasons from 2017 to 2019. The top 20 cm of soil in the experimental paddy field contained 31.7 g kg^−1^ organic matter, 1.2 g kg^−1^ total nitrogen, 0.9 g kg^−1^ total phosphorus, and 18.5 g kg^−1^ total potassium. In addition, it had 113.3 mg kg^−1^ available N, 18.6 mg kg^−1^ available phosphorus, 97.4 mg kg^−1^ available potassium, and a pH of 5.5. Three N rates (90, 150, and 210 kg ha^−1^, denoted N_90_, N_150_, and N_210_) were used in the main plots, and four plant densities (9.0, 13.5, 18.0, and 22.5 hills per m^2^, denoted D_1_, D_2_, D_3_, and D_4_) were used in 2017. Nitrogen was applied in three treatments: 60% at basal/bud sprouting, 20% at tillering, and 20% at the panicle initiation stage. Superphosphate (for phosphorus (P)) and potassium chloride (for potassium (K)) were applied at 60 kg P_2_O_5_ ha^−1^ and 60 kg K_2_O ha^−1^. P was applied at basal/bud sprouting, and K was applied in two treatments: 50% at basal/bud sprouting and 50% at the panicle initiation stage. In 2018 and 2019, sprouting fertilizer, tillering fertilizer, and panicle initiation fertilizer were applied on 25 and 28 March, 8 and 10 April, and 12 and 14 May, respectively. In 2018 and 2019, the fertilizer N:P:K ratio and corresponding management practices were the same as those in the first season of 2017. Each year, a dose of 225 kg ha^−1^ urea was applied as a grain filling fertilizer after the first season of rice full heading. The distance between the main plots was 80 cm, and the distance between the subplots within a group was 35 cm. The main treatments were separated by a robust plastic sheet measuring 65 cm high and inserted 30 cm deep into the mud to minimize seepage between plots. In 2017, pre-germinated seeds were sown on 5 March, and seedlings at the 4.5-leaf stage were transplanted on 10 April, with two seedlings per hill. The rice growth cycle for each year comprised the first season of harvested grains and the second season of harvested ratoon forage. Three seasons of rice grain and ratoon forage were harvested from 2017 to 2019. Details on the sowing, transplanting/sprouting, full heading, and ripening dates and growth duration of the perennial rice over six seasons from 2017 to 2019 are presented in Table 1.

#### 4.1.2. Experiment 2: Effect of Stubble Height and Water Management on Overwintering Seedling Development of Perennial Rice

A two-factor randomized block design experiment was conducted from June 2017 to November 2019, with two water management practices during the perennial rice’s overwintering period (shallow water irrigation (3–4 cm) and alternating dry and wet water management) and four levels of stubble heights (0, 10, 20, 30, and 40 cm) in triplicate. Plots were arranged in a split-plot design, with the water management practice examined in the main plot and the stubble height examined in the subplots. The main treatments were separated by a robust plastic sheet measuring 65 cm high and inserted 30 cm deep into the mud to minimize seepage between the plots. Pre-germinated seeds were sown on 5 June, twenty-day-old seedlings were transplanted at a spacing of 30 cm × 20 cm with four seedlings per hill on 25 June, and rice plants were harvested on 12 October 2017. In 2018 and 2019, the sprouting, first-season ripening, and harvesting dates of the ratoon forage of the perennial rice were 26 and 29 March, 22 and 24 August, and 20 and 21 November, respectively. Urea was used as a source of N, single superphosphate was used as a source of P, and potassium chloride was used as a source of K, with rates of 150 kg N ha^−1^, 75 kg P_2_O_5_ ha^−1^, and 180 kg K_2_O ha^−1^. Nitrogen was applied in three treatments: 60% at basal/bud sprouting, 20% at tillering, and 20% at the panicle initiation stage. P was used at basal, and K was applied in two treatments: 50% at basal and 50% at the panicle initiation stage. Each year, a dose of 225 kg ha^−1^ urea was used as a grain filling fertilizer after the full heading of the main rice crop. The rice plants were harvested on 12 October 2017, with a yield of 8439.0 kg ha^−1^.

#### 4.1.3. Experiment 3: Effect of Soil Moisture Content on Overwintering Seedling

##### Development and Uniformity

A field experiment beginning in June 2018 and ending in November 2020 was conducted. Four drought treatments with a duration of 30, 60, 90, and 120 days (denoted D_30_, D_60_, D_90_, and D_120_) were carried out during the perennial rice’s overwintering period (maintaining shallow water at 3 to 5 cm after the end of the drought treatment), with natural rainfall during the drought treatment period. Pre-germinated seeds were sown on 5 June, and the seedlings were transplanted on 23 June 2018, with four seedlings per hill according to the prior specification of 30 cm × 20 cm. The rice crops were harvested on 20 October 2018, drained, and allowed to naturally dry until November. For the control (CK), shallow water of 3 to 5 cm depth was maintained throughout the experiment, with five treatments. Plots were 20 m^2^ in size and replicated three times. The inter-row and inter-plot passages were 40 cm, separated by robust plastic sheets for field experiments. In 2019 and 2020, the sprouting, first-season ripening, and harvesting dates of the ratoon forage of the perennial rice were 25 and 27 March, 21 and 23 August, and 22 and 23 November, respectively.

#### 4.1.4. Experiment 4: Integrated Technique for Perennial Rice in Field Demonstration

The field study using Lunuo 1 was performed in Lu County. Pre-germinated seeds were sown on 5 June 2021; sixteen-day-old seedlings were transplanted at a 30 cm × 20 cm spacing, with four seedlings per hill, on 22 June 2021. The rice plants were harvested on 20 October 2021. In 2022, the sprouting, first-season ripening, and harvesting dates of the ratoon forage of the perennial rice were 24 March, 19 August, and 18 November, respectively. The fertilizer management practices were the same as those in experiment 2. Three treatments were applied:

1. The overwintering whole seedling technique (OWST): Rice stubble was left at a height of 10–12 cm; water management began with natural drainage for 90 days starting on 1 November 2021, and shallow water (about 4 cm) was then applied after 1 February 2022.
2. Overwintering local technology (CK_1_): Rice stubble was maintained at a height of 30–33 cm; water management involved maintaining a shallow level (3–4 cm) from 20 October 2021 to July 2022, following harvesting of the rice plants.
3. The traditional technique (CK_2_): Pre-germinated seeds were sown on 5 March 2022, and thirty-day-old seedlings were transplanted at a spacing of 30 cm × 20 cm, with five seedlings per hill, on 17 April 2022.

### 4.2. Measurements

#### 4.2.1. Growth Duration and Regrowth Tiller Development and Growth

The dates of sowing/sprouting, transplanting, heading, and maturity (harvesting date) were recorded from 2017 to 2022. Ten days after the transplanting/sprouting stage, 40 hills in each plot were marked to count the tillers (including the main stems) at 5-day intervals until the number decreased. Tillers with at least one visible leaf were counted. The regrowth rate, sprouting bud stem rate, and regrowth bud sprouting index were calculated using the following formulas:

Regrowth rate (%) = Regrowth hills number/Total hills number × 100(1)

Sprouting bud stem rate (%) = Stem number of buds sprouting/Total overwintering stems number × 100(2)

Seedling development index = Regrowth rate/Sprouting bud stem rate(3)

#### 4.2.2. Grain Yield and Yield Components

At the maturity stage in the first season of each year, 30 hills were selected for counting to determine the number of panicles per unit of land area. Six hills were sampled for each plot, after counting the number of panicles, all plants were separated into straw and grains. Filled spikelets were separated from unfilled spikelets by submerging in tap water. After oven-drying at 70 °C to a constant weight, the dry weights of the straw, filled spikelets, and unfilled spikelets were determined. Three subsamples of 30 g filled spikelets and all unfilled spikelets were taken to calculate the grain weight, grain filling percentage, and spikelets per panicle. Grain yield was determined from a 5 m^2^ area in the middle of each plot and adjusted assuming a standard moisture content of 135 mg H_2_O g^−1^.

#### 4.2.3. Regenerated Forage Yield and Its Nutrients

In November of each year, ratooning forage yield was determined from a 5 m^2^ area in the middle of each plot after oven-drying at 70 °C to a constant weight. The crude protein, crude fat, crude fiber, calcium, nitrogen, phosphorus, and potassium contents of the ratoon forage were determined. After counting the number of panicles, twenty hills were sampled for each plot, and the plants were separated into straw, fertilized grains, and unfertilized grains. The dry weight of each plant organ was determined after oven-drying at 70 °C to a constant weight. The grain filling percentage, grain plumpness, and grain plumpness index were calculated using the following formulas:

Percentage of grain filling (%) = Total number of filled grains per panicle/Total number of grains per panicle × 100(4)

Grain plumpness (%) = Average fertilized grain weight/Full grain weight × 100(5)

Index of grain plumpness = Percentage of grain filling/Grain plumpness(6)

### 4.3. Statistical Analysis

Climatic data for this study were obtained from local meteorological bureaus. Statistical analyses were conducted using Statistix 8 software (Analytical Software, Tallahassee, FL, USA) and analysis of variance (ANOVA). For experiment 1, the ANOVA model included the following factors: replication; year (Y); nitrogen application rate (N); planting density (PD); and the interactions between these factors, specifically Y × N, Y × PD, and PD × N and the three-factor interaction of Y × N × PD. For experiment 2, the ANOVA model included the following factors: replication; year (Y); water management practice (W); stubble height (H); and the interactions between these factors, specifically Y × W, Y × H, and W × H and the three-factor interaction of Y × W × H. For experiment 3, the ANOVA model included the following factors: replication, year (Y), drought treatment (D), and the interaction of Y × D. The criterion for statistical significance was set at the 0.05 probability level. 

## 5. Conclusions

The perennial rice variety Lunuo 1 was successfully overwintered in Luxian County; moreover, it could be harvested twice in one year, with rice grains harvested in the first season and ratoon forage grass harvested in the second season. The grain and ratoon forage yields of the perennial rice decreased with each successive regrowth season. Still, both the grain and ratoon forage yields of the perennial rice increased with increases in the N rate and planting density. Both the grain and ratoon forage yields could be enhanced by increasing the regrowth rate through optimizing the rice stubble height, soil moisture, and water management during the winter season. Our results suggest that integrating N, water, and other management practices (including the combination of a 150 kg ha^−1^ N rate, 18.0 hills per m^2^, 10–20 cm rice stubble height, and alternating dry and wet irrigation during the winter season) is a feasible approach for achieving both high grain and ratoon forage yields in perennial rice systems.

## Figures and Tables

**Table 1:** Dates for sowing, sprouting, full heading, ripening, and growth duration of perennial rice over six seasons from 2017 to 2019.

Cropping System (Year)	Sowing/Sprouting (Day/Month)	Full Heading (Day/Month)	Ripening (Day/Month)	Growth Duration (Day)
First season (2017F)	5/3	5/7	11/8	159
Second season (2017S)	23/8	26/9	12/11	81
Third season (2018F)	25/3	7/7	20/8	148
Fourth season (2018S)	25/8	28/9	18/11	85
Fifth season (2019F)	28/3	9/7	25/8	150
Sixth season (2019S)	26/8	30/9	21/11	87

**Table 2:** Effects of N rate and planting density on regrowth rate (%), panicles per m^2^, spikelets per panicle, grain filling (%), grain weight (mg), and grain yield (kg ha^−1^) in the perennial rice system from 2017 to 2019.

Treatment	Regrowth Rate (X_1_, %)	Panicles per m^2^ (X_2_)	Spikelets per Panicle (X_3_)	Grain Filling (X_4_, %)	Grain Weight (X_5_, mg)	Grain Yield (kg ha^−1^)
Year (Y)						
2017F	100.00 c	281.65 a	158.69 c	84.19 a	23.50 b	8394.49 a
2018F	81.98 b	186.86 b	170.75 a	83.65 a	23.83 a	6029.04 b
2019F	54.12 a	111.18 c	163.73 b	83.84 a	23.38 b	3289.40 c
N rate (kg ha^−1^)						
90	78.63 a	182.65 c	168.72 a	84.47 a	23.65 a	5808.01 b
150	78.62 a	194.28 b	165.42 b	83.32 a	23.48 a	5893.84 ab
210	78.85 a	202.76 a	159.03 c	83.89 a	23.57 a	6011.08 a
Planting density (hills per m^2^)						
9.0	77.98 a	159.87 d	177.06 a	83.96 a	23.54 a	5319.65 c
13.5	78.32 a	185.78 c	167.52 b	83.58 a	23.44 a	5713.27 b
18.0	79.00 a	206.18 b	160.28 c	84.45 a	23.69 a	6284.55 a
22.5	78.62 a	221.08 a	152.70 d	83.58 a	23.61 a	6299.77 a
Analysis of variance						
Year (Y)	**	**	**	ns	**	**
N rate (N)	ns	**	**	ns	ns	*
Planting density (PD)	ns	**	**	ns	ns	**
Y × N	ns	ns	*	ns	ns	ns
Y × PD	**	**	**	ns	ns	*
N × PD	ns	ns	ns	ns	ns	ns
Y × N × PD	ns	ns	ns	ns	ns	ns

Note: Different lowercase letters in the same column indicate significant differences at the 0.05 level. * and ** denote significant differences at the 0.05 and 0.01 probability levels, respectively, as determined using ANOVA. ns denotes non-significance based on ANOVA.

**Table 3:** Contribution of regrowth rate, number of panicles, number of spikelets per panicle, grain filling, and grain weight to grain yield of first-season rice from 2017 to 2019.

Factor	Correlation	Direct Contribution	Indirect Contribution					
			Total	X_1_	X_2_	X_3_	X_4_	X_5_
X_1_	−0.9786 **	−0.0991	−0.8795		−0.8895	0.0270	−0.0053	−0.0117
X_2_	0.9678 **	0.9628	0.0051	0.0916		−0.0972	0.0040	0.0067
X_3_	−0.2858	0.1983	−0.4841	−0.0135	−0.4720		−0.0029	0.0043
X_4_	0.1353	0.0496	0.0857	0.0105	0.0772	−0.0116		0.0096
X_5_	0.2463	0.0441	0.2023	0.0264	0.1455	0.0195	0.0109	

Note: X_1_, X_2_, X_3_, X_4_, and X_5_ represent the regrowth rate, number of panicles, number of spikelets per panicle, grain filling, and grain weight, respectively. ** denotes significant correlation coefficients at the 0.01 probability level.

**Table 4:** Effect of average daily temperature after ratoon rice full heading on percentages of grain filling, grain plumpness, and index of grain plumpness from 2017 to 2019.

Year	Average Daily Temperature after Heading in the Ratoon Forage Season (°C)					Grain Filling (%)	Grain Plumpness (%)	Index of Grain Plumpness (%)
	1–10 d	11–20 d	21–30 d	31–40 d	Average			
2017	21.4	18.1	17.3	17.0	18.5	28.73 a	38.66 a	11.11a
2018	18.0	16.8	16.8	16.6	13.3	26.68 b	31.09 b	8.29 b
2019	21.7	19.0	16.6	16.6	18.5	29.71 a	39.82 a	11.83 a
Average	20.4	18.0	16.9	16.7	16.8	28.37	36.52	10.41

Note: Different lowercase letters in the same column indicate significant differences up to the 0.05 level.

**Table 5:** Effects of N rate and planting density on ratoon forage yield and its feed quality in the perennial rice system from 2017 to 2019.

Treatment	Regrowth Rate (%)	Ratoon Forage (kg ha^−1^)	Total N (g/100 g)	Crude Protein (g/100 g)	Crude Fat (g/kg)	Crude Fiber (g/100 g)	Calcium (g/100 g)	Phosphorus (g/100 g)	Potassium (g/100 g)
Year									
2017S	97.23 a	5663.78 a	0.78 a	4.96 a	16.89 a	23.75 a	0.27 a	0.16 a	1.06 a
2018S	75.27 b	4021.00 b	0.76 b	4.66 b	16.72 a	21.95 b	0.25 b	0.15 a	0.93 b
2019S	46.57 c	2676.64 b	0.72 c	4.43 c	15.35 b	20.87 c	0.25 b	0.14 b	0.88 c
N rate (kg ha−1)									
90	72.95 a	3902.24 b	0.70 c	4.32 c	15.30 c	22.15 b	0.25 b	0.15 b	0.95 b
150	72.92 a	4412.35 a	0.77 b	4.74 b	16.12 b	21.85 b	0.25 b	0.15 b	0.93 b
210	73.23 a	4046.83 b	0.79 a	5.00 a	17.54 a	22.58 a	0.27 a	0.16 a	0.99 a
Planting density (hills per m^2^)									
9.0	73.82 a	3832.90 c	0.74 b	4.54 b	15.57 c	21.84 b	0.24 c	0.15 a	0.97 a
13.5	72.12 a	4225.02 ab	0.75 ab	4.67 ab	16.14 b	22.45 a	0.26 b	0.15 a	0.95 ab
18.0	73.50 a	4086.47 b	0.76 a	4.73 a	16.56 ab	22.30 a	0.25 b	0.15 a	0.92 b
22.5	72.70 a	4337.50 a	0.77 a	4.80 a	17.01 a	22.17 ab	0.28 a	0.16 a	0.99 a
Analysis of variance									
Year (Y)	**	**	**	**	**	**	**	**	**
N rate (N)	ns	**	**	**	**	**	**	**	**
Planting density (PD)	ns	**	*	*	**	*	**	ns	*
Y × N	ns	*	ns	ns	ns	**	ns	ns	**
Y × PD	**	ns	ns	ns	ns	ns	ns	ns	ns
N × PD	ns	*	ns	ns	ns	**	**	ns	ns
Y × N × PD	ns	ns	ns	ns	ns	ns	ns	ns	ns

Note: Different lowercase letters in the same column indicate significant differences up to the 0.05 level. * and ** denote significant differences at the 0.05 and 0.01 probability levels, respectively, as determined using ANOVA. ns denotes non-significance based on ANOVA.

**Table 6:** Effect of stubble height and water management practices on regrowth rate and maximum tillers in the perennial rice system from 2018 to 2019.

Year	Stubble Height (cm)	Regrowth Rate (%)		Maximum Tiller (Tillers per Hill)	
		Shallow Irrigation	Dry and Wet Alternation	Shallow Irrigation	Dry and Wet Alternation
2018	0	0.00 b	0.00 d	0.00 c	0.00 d
	10	73.35 a	100.00 a	10.41 a	16.64 a
	20	74.22 a	100.00 a	10.68 a	17.38 a
	30	75.15 a	97.29 b	7.14 b	13.91 b
	40	74.53 a	94.71 c	7.62 b	11.27 c
2019	0	0.00 b	0.00 d	0.00 c	0.00 d
	10	80.37 a	100.00 a	12.64 a	18.32 a
	20	81.16 a	100.00 a	12.91 a	19.15 a
	30	81.11 a	95.24 b	9.57 b	15.77 b
	40	82.51 a	96.65 c	8.80 b	12.03 c
Analysis of variance					
Year (Y)		*		**	
Water management (W)		**		**	
Stubble height (H)		**		**	
Y × W		*		**	
Y × H		ns		**	
W × H		**		**	
Y × W × H		**		**	

Note: Different lowercase letters in the same column indicate significant differences up to the 0.05 level. * and ** denote significant differences at the 0.05 and 0.01 probability levels, respectively, as determined using ANOVA. ns denotes non-significance based on ANOVA.

**Table 7:** Effects of soil moisture content on overwintering seedling development index and yield.

Year	Drought (Days)	Relative Water Content (%)	Regrowth Rate (%)	Sprouting Bud Stem Rate (%)	Seedling Development Index (%)	Initiation Heading to Full Heading (Days)	First-Season Yield (kg/ha)	Second-Season Forage Yield (kg ha^−1^)
2019	CK	99.99 a	77.31 d	68.24 d	52.76 d	22.36 c	4929.30 d	5376.90 d
	D_30_	92.36 b	82.65 c	76.89 c	63.55 c	19.72 b	6625.95 c	5592.15 c
	D_60_	85.22 c	92.87 b	81.06 b	75.28 b	16.94 a	7788.90 b	6278.25 b
	D_90_	78.85 d	99.34 a	85.44 ab	84.83 a	14.68 a	8612.25 a	6845.40 a
	D_120_	67.91 e	100.00 a	86.48 a	86.48 a	14.85 a	8546.70 a	6900.60 a
2020	CK	99.99 a	83.17 d	71.43 d	59.41 d	20.57 c	4608.45 d	4906.05 d
	D_30_	88.45 b	86.26 c	79.18 c	68.30 c	17.33 b	6201.91 c	5208.90 c
	D_60_	81.31 c	95.87 b	85.67 b	82.13 b	14.56 a	7300.05 b	5976.75 b
	D_90_	74.77 d	100.00 a	88.03 ab	88.03 a	13.94 a	8296.24 a	6540.45 a
	D_120_	68.84 e	100.00 a	91.92 a	91.92 a	13.67 a	8227.82 a	6772.65 a
Analysis of variance								
Year (Y)		ns	ns	**	**	**	**	**
Drought treatment (D)		**	**	**	**	**	**	**
Y × D		ns	ns	**	**	**	**	**

Note: Different lowercase letters in the same column indicate significant differences up to the 0.05 level. ** denote significant differences at the 0.05 and 0.01 probability levels, respectively, as determined using ANOVA. ns denotes non-significance based on ANOVA.

**Table 8:** Comparison of regrowth rate, grain yield, panicles per m^2^, spikelets per panicle, grain filling, grain weight, and ratoon forage yield integrated technique for perennial rice (2022).

Treatment	Regrowth Rate (%)	Panicles per m^2^	Spikelets per Panicle	Grain Filling (%)	Grain Weight (mg)	Grain Yield (kg/hm^2^)	Ratoon Forage Yield (kg ha^−1^)
OWST	99.17	288.60	153.82	85.70	23.69	8495.43	7310.74
CK1	70.29	234.15	148.56	85.71	23.62	6489.75	5496.32
CK2	100.00	309.15	142.56	85.21	23.59	8631.45	7383.75

OWST: overwintering whole seedling technique (OWST); CK1: overwintering local technology; CK2: local conventional technique.

**Table 9:** Average daily temperature (ADT), average maximum temperature (AMT), average minimum temperature (AMT_1_), duration of sunshine hours (DSH), total precipitation (TP), and average relative humidity (ARH) from 2017 to 2021.

Year	ADT (°C)	AMT (°C)	AMT_1_ (°C)	DSH (h)	TP (mm)	ARH (%)
2017	18.8	22.8	16.0	1232.0	878.1	82.9
2018	18.7	22.8	15.8	1358.2	1004.3	83.1
2019	18.3	22.2	15.8	983.1	1185.6	81.9
2020	18.5	22.4	15.9	1050.2	1446.6	79.8
2021	18.6	22.6	15.9	1038.1	1018.8	80.8
2022	19.8	23.8	16.7	1429.0	1138.0	77.8

## Data Availability

Data are contained within this article.
